# Japanese translation and modification of the Oslo Sports Trauma Research Centre overuse injury questionnaire to evaluate overuse injuries in female college swimmers

**DOI:** 10.1371/journal.pone.0215352

**Published:** 2019-04-15

**Authors:** Yasuharu Nagano, Keisuke Kobayashi-Yamakawa, Ayako Higashihara, Hiroko Yako-Suketomo

**Affiliations:** 1 Department of Sports Wellness Sciences, Japan Women’s College of Physical Education, Tokyo, Japan; 2 Faculty of Sport Sciences, Waseda University, Satiama, Japan; São Paulo State University (UNESP), BRAZIL

## Abstract

The purpose of the present study was to translate and modify the Oslo Sports Trauma Research Centre (OSTRC) overuse injury questionnaire into Japanese and validate it among Japanese athletes through a longitudinal survey. A modified back-translation method was used to translate the questionnaire from English to Japanese. The longitudinal survey was performed in 29 female college swimmers who were followed up for more than 24 consecutive weeks. The response rate to the 24 weekly questionnaires was 88.8% (95% confidence interval [CI]: 85.2–92.3). Internal consistency was measured by using Cronbach’s alpha (0.73 (0.69–0.77)). The anatomical areas most frequently affected by overuse injuries were the lower back (average weekly prevalence: 27.6%, 95% CI: 25.1–30.1), shoulder (16.0%, 95% CI: 13.7–18.2), knee (9.9%, 95% CI: 7.7–12.0), and ankle (9.0%, 7.6–10.5). The severity score showed that knee (22.5, range: 6–65), ankle (21.5, range: 6–67), and lower back (20.7, range: 6–80) injuries had the greatest impact. The Japanese version of the modified OSTRC overuse injury questionnaire demonstrated reliability and validity based on the results of internal consistency and trend of injury of the swimmers. The participants in the present study did not have substantial injuries or time-loss injuries and continued practicing and competing, despite these minor injuries. Although knee and ankle injuries do not occur as often as lower back and shoulder injuries, these injuries often had a greater impact on swimmers when they did occur.

## Introduction

Recently, injury surveillance has been conducted to better understand sporting injury incidence during activities and to examine prevention methods. However, the results of these injury incidences have changed greatly according to the definition of injury. There are three different definitions of injury: ‘any physical complaint’, ‘medical attention injury’, and ‘time-loss injury’.[1, 2] The definition of injury will influence the rates of injury that are reported in studies because players will not always seek medical attention for physical complaints, and even fewer athletes will experience time-loss injuries.[1] The ‘time-loss’ injury is the definition most frequently used [1] and is suitable for many sports in which acute injuries lead to time loss during games and practice. However, in some sports such as swimming, which require endurance or technical skill, a time-loss injury is rare and therefore the ‘time-loss’ definition is not appropriate. For example, Yang et al.[3] reported the epidemiology of overuse and acute injuries among college athletes according to the ‘time-loss’ definition. Female swimmers demonstrated a comparable rate when overuse and acute injuries were evaluated (9.7/10000 AEs and 8.5/10000 AEs, respectively).[3] Kerr et al.[4] reported the epidemiology of injuries in college athletes in swimming according to the ‘medical attention’ definition. Female swimmer overuse injuries (63.7%) were shown to be higher than that of the other types of injuries.[4] If the definition of ‘any physical complaint’ is applied, the number and rate of overuse injuries are likely to be higher. However, these data according to the definition of ‘any physical complaint’ have not been reported. Additionally, overuse injuries generally occur gradually, whereas acute injuries occur suddenly thereby directly influencing performance or participation. Therefore, detecting overuse injury in the early stages is important to prevent worsening of the condition and “time-loss” injury.

In 2013, Clarsen et al.[5] developed and validated a new questionnaire to register overuse injuries in sports: the Oslo Sports Trauma Research Centre (OSTRC) overuse injury questionnaire. This questionnaire collects data from the athletes regarding pain, limited participation in training and competition, and reduced training volume and performance capacity in sports and allows the evaluator to calculate the prevalence and severity score of overuse injuries that affect a specific anatomical area.[5] The questionnaire compares the prevalence of overuse injuries among athletes.[6] This questionnaire is useful to register and monitor the overuse injury according to the ‘any physical complaint’ definition, therefore the OSTRC overuse injury questionnaire was translated into Danish,[7] and Swedish [8] for international comparison. Clarsen et al. [9] also developed another questionnaire (the OSTRC questionnaire on health problems) to monitor the health conditions of the Olympic athletes. This questionnaire on health problems was translated into German,[10] and Danish.[7] These methods are becoming the global standard of sports medicine to capture the severity of overuse injuries.

In contrast, no tools in the Japanese language are available to capture data on all overuse injuries based on the ‘any physical complaint’ definition. Therefore, the purpose of the present study was to translate and modify the OSTRC overuse injury questionnaire into Japanese. Furthermore, the goal was to validate the questionnaire in Japanese college swimmers as a model for which the ‘any physical complaint’ definition can be applied. Although there is the OSTRC questionnaire on health problems, we selected to translate the OSTRC overuse injury questionnaire to examine the prevalence and impact of overuse injury in the present study.

## Materials and methods

### Ethical considerations

This prospective study was approved by the Ethics Committee of the Japan Women's College of Physical Education (approval number: 2015–24) and complied with the ethical principles of the Declaration of Helsinki. All athletes provided written informed consent prior to participating in the study.

### The questionnaire

The questionnaire that was used in the present study was developed in Norway at the OSTRC.[5] The OSTRC Overuse Injury Questionnaire contains four key questions recording the level of participation and performance impairment and the degree of symptoms of an injury or illness.

### Translation procedure

The English version of the OSTRC Overuse Injury Questionnaire[5] was used for the Japanese translation. The translation of the questionnaire was conducted using the back-translation method presented by Werner and Campbell. [11] This process included the following four steps, referring to a previous study.[8]

#### Steps 1 and 2: Back-translation

The forward translation from English to Japanese was performed by a Japanese physiotherapist who has a Ph.D. and specializes in sports medicine, whose native language is Japanese, but second language is English. The back-translation was performed by a specialized supplier (Translation agency: Ulatus, Crimson Interactive Pvt. Ltd.). In this step, a translator (TL1) performed the back-translation without looking at the original version and translated the Japanese version back into English. Subsequently, another translator (TL2) compared the original and back-translated English versions and noticed the difference in terms and expressions to inspect the accuracy of the Japanese version. The translator (TL2) reported the results of the inspection. Finally, the translator (TL2) corrected and completed the Japanese version.

#### Step 3: Discussion by an expert committee

**Fig 1:**
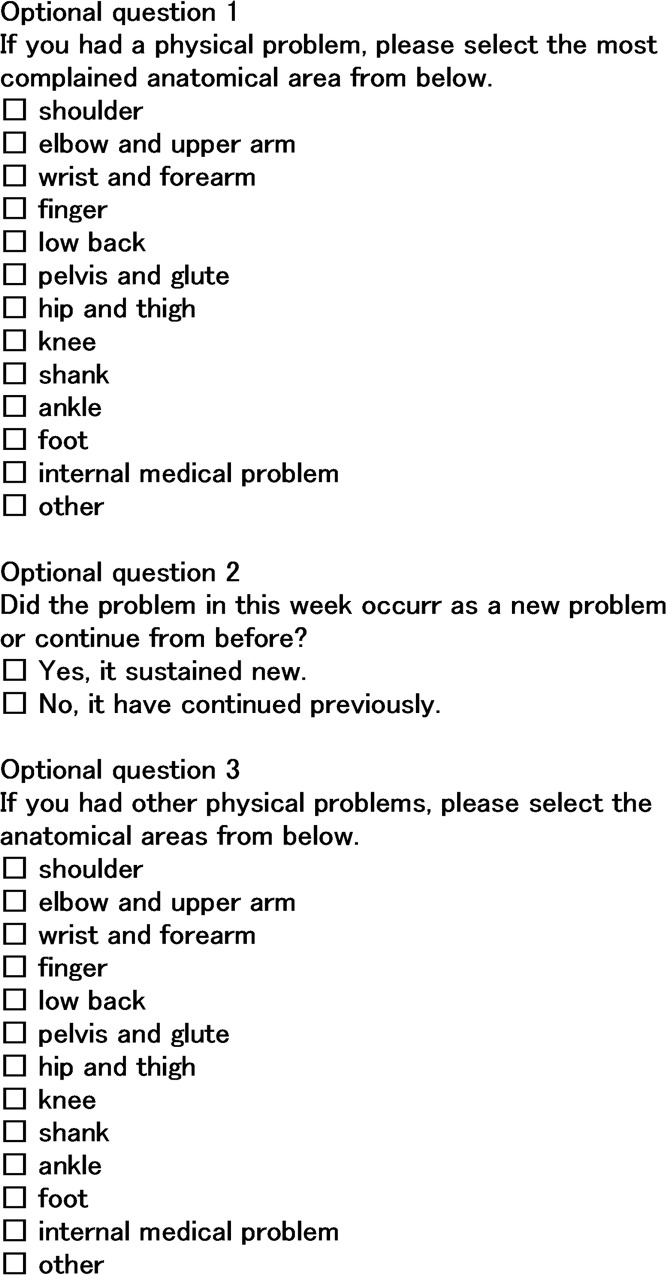
The list of three additional questions.

An expert committee that included a physiotherapist who was involved in the translation, another physiotherapist, one researcher who specialized in sports medicine, and one researcher who specialized in health promotion collaborated to evaluate the questionnaire. No discrepancies regarding the use of synonyms or use of grammar were noted by the expert committee. The expert committee agreed to change the method of responding to questions on the anatomical areas. The original version consisted of 12 initial questions, because three body areas were examined. In the Japanese version that was used in the present study, there were four main questions for all physical complaints, with another question that asked about the anatomical areas that were complained about the most, to decrease the time it took the participant to answer the questionnaire and increase the response rate. The anatomical areas consisted of the shoulder, elbow and upper arm, wrist and forearm, fingers, lower back, pelvis and gluteus, hip and thigh, knee, shank, ankle, and foot, and we also asked about internal medical problems. If a respondent had other problems that affected training and competitions, they could answer the optional question regarding other anatomical areas of concern. Additionally, the expert committee agreed to add the question: ‘Did the problem this week occur as a new problem or did it continue from before?’ The final Japanese version of the questionnaire contained four multiple-choice questions for all physical complaints and the three additional questions followed, where the participants reported the problems (Fig 1). The time needed to complete the questionnaire was estimated as 1–2 min.

#### Step 4: Pretest

We conducted a pretest to evaluate any deficit in the Japanese version of the questionnaire for athletes. The pretest was administered five times every 4 weeks to 30 female college swimmers who were inter-college to district-level athletes. During and after the pretest, we interviewed the participants regarding the ease of answering the questions and unclear questions. The questionnaire was not modified in this step, because there was no demand for it. The translation procedure was then subsequently completed.

### Longitudinal survey setting

We conducted a longitudinal survey of 29 female college swimmers who were inter-college to district-level athletes. When we set the expected proportion of prevalence as 0.5, total width of confidence interval as 0.4, and level of confidence as 95%, the required number of participants was 24. [12] We selected female swimmers as subjects because they often suffer from the type of overuse injuries mentioned in the introduction section. The Japanese version of the OSTRC questionnaire was distributed weekly to all athletes electronically for 24 weeks using the Google form platform. An internet link was distributed to the athletes via a message application. If the participants reported that they had any recent health problems within 1 month, they were contacted by a physiotherapist to determine the type and nature of each problem. Based on these interviews, all injuries were classified as acute or overuse injuries. Acute injuries were defined as those associated with a specific, clearly identifiable injury event.[6] All other injuries were considered overuse injuries, regardless of whether their onset was gradual or rapid. The injury which classified acute injury was excluded from the results of this study.

### Prevalence measures

The prevalence of overuse injuries was calculated for all overuse injuries as well as each anatomical area during each week of the study period by dividing the number of athletes who reported any problems, by the number of questionnaire respondents.[6] For each anatomical area, the numerator in the prevalence calculation included the number of people who responded to the anatomical area as being the chief complaint area and those who reported it as an additional complaint. Weekly prevalence data were plotted over time to identify trends over the course of the study period, and the average weekly prevalence of overuse injuries was calculated for each anatomical area. The average prevalence of substantial overuse injuries was also calculated. The numerator for the substantial overuse injuries of each anatomical area was the number of people who reported it as the chief complaint area. The numerator in the prevalence calculations of substantial injuries only included overuse injuries leading to self-reported moderate or severe reductions in training volume or sporting performance, or a complete inability to participate (i.e., responses of c, d, or e in either question 2 or 3).[6] Every time an athlete responded to the questionnaire, a severity score for the most complained anatomical area was calculated, based on their responses to the four key questions [5]: “The responses to each of the four questions were allocated a numerical value from 0 to 25, and these were summed in order to calculate a severity score from 0 to 100 for each overuse problem. The response values were allocated such that 0 represented no problems and 25 represented the maximum level for each question. The values for intermediate responses were chosen in order to maintain as even a distribution from 0 to 25 as possible while still using whole numbers. Therefore, questions 1 and 4 were scored 0-8-17-25, and questions 2 and 3 were scored 0-6-13-19-25. The average severity score for each anatomical area was also calculated over the 24 weeks of the study period.

### Statistics

The average weekly prevalence of all overuse injuries and substantial overuse problems are expressed as averages and 95% confidence intervals (95% CI). The severity score for all overuse injuries were expressed as average weekly and range. The severity scores for injuries of each anatomical area through research period were expressed as the average of all reported injuries in each area and range. Internal consistency was determined by calculating the Cronbach’s alpha value for all complete questionnaire responses, with 0 indicating no internal consistency and 1 corresponding to perfect internal consistency. The Cronbach’s alpha of all reported questionnaires was calculated for the four key questions. The inter-item correlation matrix was assessed to determine whether it would be necessary for the questionnaire. The intraclass correlation coefficients (ICC) for the severity score were calculated from the data 4 times from the start of the research to show single measures [ICC (1, 1)] and absolute agreement [ICC (1, 4)] of a month.

## Results

### Translation and adaptation

In the steps of back-translation, an omission of the translation was found, which is “without XX problems” in Q1. Therefore, the Japanese equivalent to this phrase was added. There was no difference in meaning between the forward translation and back-translation versions. During discussion by the expert committee, the method of responding to questions on the anatomical areas was changed. As a result, the Japanese version resembled closely the questionnaire of health problems. [9] This modification and reconstruction of the questionnaire was approved by the authors of the original version[5].

Cronbach’s alpha was 0.78, when all the questionnaires were analysed. (Table 1) Owing to no further change in Cronbach’s alpha following removal of items in either case, the four key questions were retained. The intraclass correlation coefficient was calculated for 14 participants who responded all 4 times from the start of the research. The ICC (1, 1) was 0.543, and ICC (1, 4) was 0.826.

**Table 1:** Inter-item correlations and effects of removing items on internal consistency.

	Interitem correlation matrix				Cronbach’s α if item deleted
	Question 1	Question 2	Question 3	Question 4	
All questionnaire (n = 567)					
Question 1	-				0.74
Question 2	0.72	-			0.79
Question 3	0.52	0.44	-		0.61
Question 4	0.37	0.29	0.79	-	0.74

### Participant characteristics

The participant characteristics are summarized in Table 2.

**Table 2:** Participant characteristics.

Participants (n)	29	(0.1)
Age (years)	19.9 ± 1.3	(0.2)
Height (cm)	161.0 ± 5.1	(0.1)
Weight (kg)	53.7 ± 5.8	(0.0)
Athletic career (years)	7.7 ± 1.2	(0.1)
Swimming strokes (n)		
Freestyle	9	
Backstroke	1	
Breaststroke	5	
Butterfly	7	
Individual medley	3	
Multiple	4	

Data are presented as mean ± standard deviation and (Coefficient of variation)

### Response rate

The total number of completed questionnaires was 567. The response rate to the 24 weekly questionnaires was 88.8% (95% CI: 84.8–92.7). The highest rate was 100%, and lowest rate was 59.3%.

### The prevalence of overuse problems

**Fig 2:**
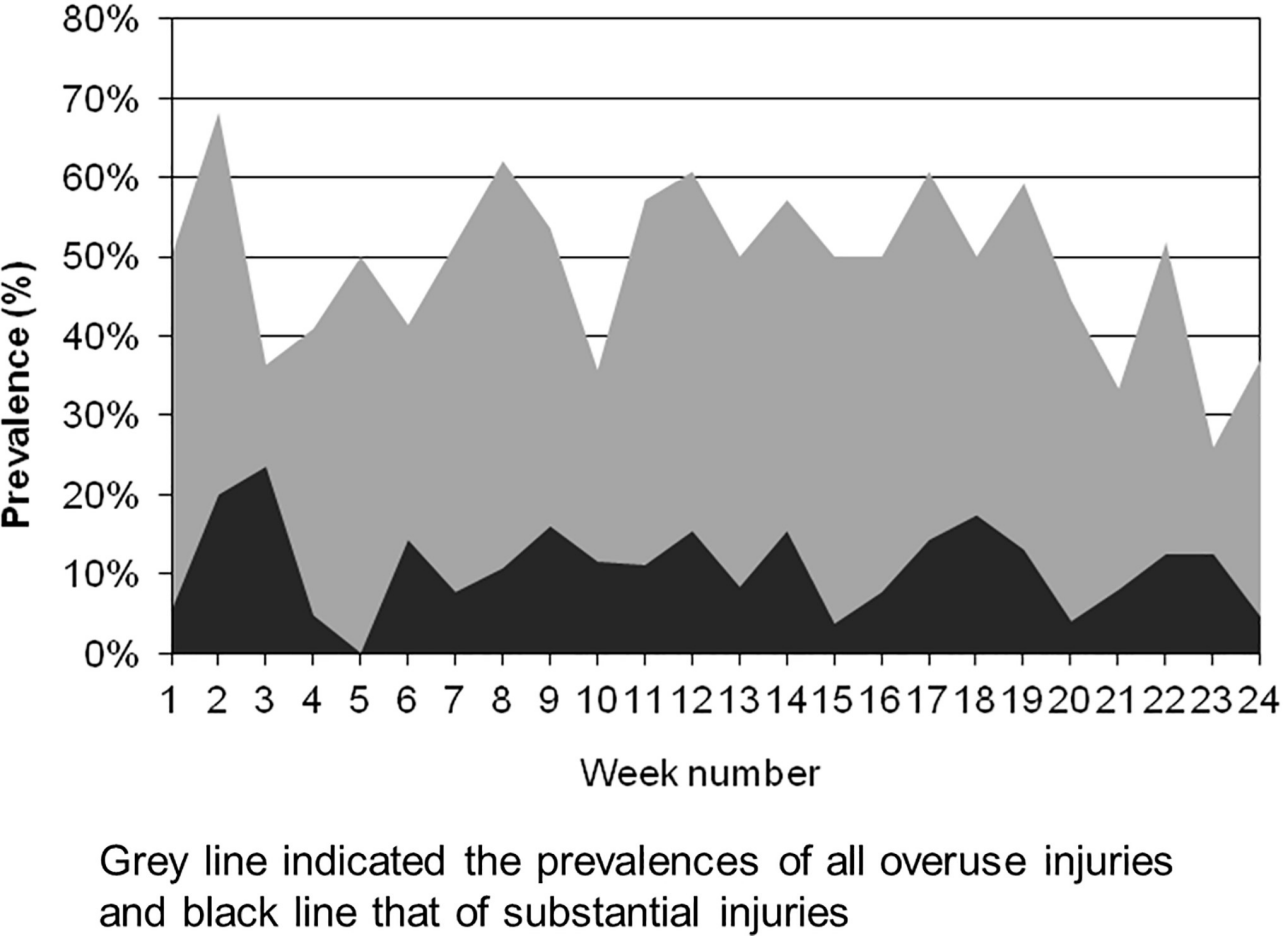
The prevalence of all overuse injuries and substantial injuries.

**Fig 3:**
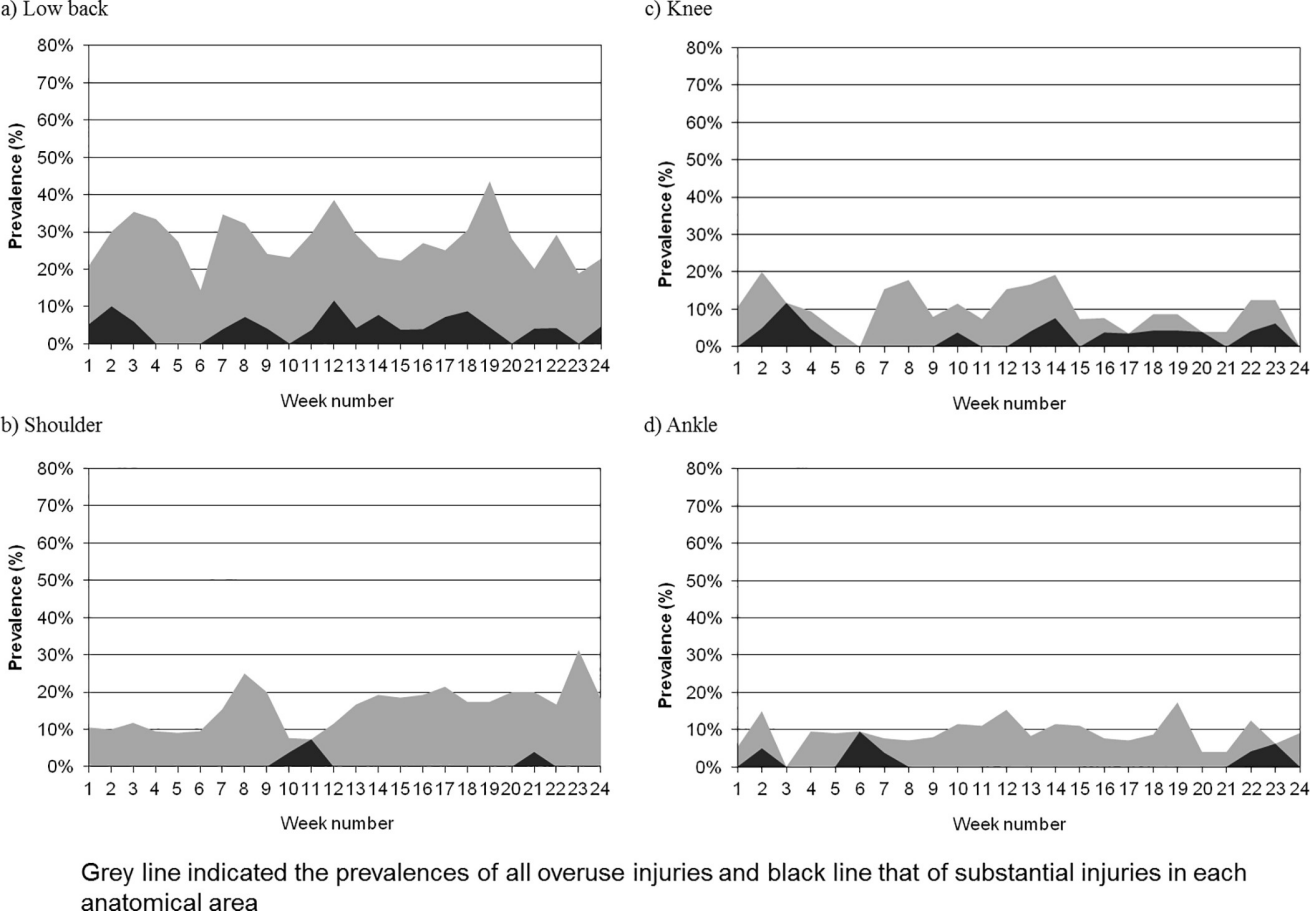
The prevalence of all overuse injuries and substantial injuries. a) lower back, b) shoulder, c) knee, and d) ankle.

Table 3 shows the average prevalence of all overuse injuries and that of substantial overuse injuries for each anatomical area. The most frequently reported problematic areas were the lower back, shoulder, knee, and ankle. The prevalence of all overuse injuries and substantial overuse injuries during the 24 weeks of the study period are illustrated in Fig 2. The prevalence fluctuated weekly. However, the prevalence of all overuse injuries and substantial injuries did not always change together. The prevalence of the lower back, shoulder, knee, and ankle overuse injuries and substantial overuse injuries during the 24 weeks of the study period are illustrated in Fig 3. The prevalence of the injuries and substantial injuries in the lower back had a tendency to change together. For the shoulder injuries, the prevalence of substantial injuries was relatively low.

**Table 3:** The average prevalence of all overuse injuries and that of substantial overuse injuries.

	All overuse injuries				Substantial overuse injuries			
Lower back	27.6	(24.8	―	30.4)	4.3	(2.9	―	5.7)
Shoulder	16.0	(13.5	―	18.5)	0.6	(-0.1	―	1.4)
Knee	9.9	(7.5	―	12.3)	2.8	(1.5	―	4.1)
Ankle	9.0	(7.4	―	10.7)	1.2	(0.1	―	2.3)
Hip / thigh	5.2	(3.0	―	7.5)	0.4	(-0.2	―	1.0)
Pelvis / glute	3.1	(1.9	―	4.3)	0.2	(-0.2	―	0.5)
Foot	2.8	(0.9	―	4.8)	0.0	(0.0	―	0.0)
Elbow / upper arm	2.2	(1.0	―	3.4)	0.2	(-0.2	―	0.6)
Internal medical problem	1.3	(0.4	―	2.3)	1.0	(0.1	―	1.9)
Wrist / forearm	1.3	(0.2	―	2.4)	0.0	(0.0	―	0.0)
Shank	1.0	(0.2	―	1.7)	0.0	(0.0	―	0.0)
Finger	0.3	(-0.1	―	0.8)	0.0	(0.0	―	0.0)
Unknown	1.0	(0.1	―	1.9)	0.1	(-0.2	―	0.5)
Total	49.1	(44.7	―	53.5)	10.9	(8.5	―	13.3)

Data are presented as percentage with 95 confidence interval

### Severity score

The weekly average severity score for all overuse injuries was 11.2 (arbitrary) and ranged 0 to 86. Table 3 shows the average severity score of overuse injuries in each anatomical area which was based on a 24-week period. As shown in Table 4, internal medical problems had a greater severity score, as well as knee, ankle, and low back problems that showed increased prevalence of injury. On the other hand, the shoulder had a relatively lower severity score for greater injury prevalence.

**Table 4:** Average severity score of injury for each anatomical area over the 24 weeks.

	Severity score (arbitrary)	Range (min―max)		
Internal medical problem	46.1	(20.0	―	86.0)
Wrist/Fore arm	23.0	(23.0	―	23.0)
Knee	22.5	(6.0	―	65.0)
Ankle	21.5	(8.0	―	67.0)
Low Back	20.7	(6.0	―	80.0)
Elbow / Upper arm	19.3	(8.0	―	44.0)
Shank	18.0	(8.0	―	28.0)
Hip / Thigh	17.7	(6.0	―	53.0)
Shoulder	16.8	(8.0	―	44.0)
Pelvis / Glute	13.4	(8.0	―	38.0)
Foot	12.4	(8.0	―	22.0)
Finger	11.0	(8.0	―	14.0)

## Discussion

The first purpose of the present study was to translate and modify the OSTRC overuse injury questionnaire that was designed to examine overuse injuries related to sporting activities, into the Japanese language. There is currently no questionnaire in the Japanese language that can be used to survey overuse injuries based on the ‘any physical complaint’ definition. We translated the original OSTRC overuse injury questionnaire from English into Japanese using a back-translation process and completed the translation process after an expert committee meeting. We changed the method of reporting the anatomical area of injury, according to the expert committee’s suggestion. Therefore, the questionnaire of the present study was different from the original version of OSTRC questionnaire of overuse injuries, which examines the occurrence and impact of injuries in particular anatomical areas. As a result, the Japanese version resembled closely the questionnaire of health problems. [9] We reconstructed this modified questionnaire and confirmed its usability. The responders did not have problems while answering the questionnaire, and the response time was short. Using an internet-based form, we could conduct the survey continuously, with a small burden on the athletes. In Japan, it might be effective to use the modified questionnaire, which combined the questionnaires of overuse injuries and health problems, to examine the all the overuse injuries based on the ‘any physical complaint’ definition.

The Japanese version of the OSTRC overuse injury questionnaire was applied to 29 female college swimmers in a longitudinal survey to examine its reliability and validity. The response rate was high throughout the research period. Most of the participants could answer it each time with only two or three replies in the questionnaire missing. These trends were consistent with those reported in previous studies.[7–10] To examine the reliability, we calculated the internal consistency as measured by the Cronbach’s alpha and ICC. The mean Cronbach's alpha over the 24-week study period was 0.73. This result was lower than that in previous studies. [6, 7, 10] When the amount of training did not change even though the athlete experienced pain, the consistency between the questions would not be high. A previous study also reported that 30% swimmers did not scale down their training regime despite symptoms. [13] Therefore, it is possible that participants of this study did not scale down the amount training as they experienced some pain. In those cases, the value of internal consistency was acceptable. On the other hand, ICC (1, 1) for the severity score was low. This was due to the change in injury condition during the first week. Before and after the first week, the condition may have been worse or better. Although the test-retest measurement in the shorter period was an option to establish the reliability, it would be difficult to implement because of the recall bias. Therefore, one of limitation of this study is that that reliability could not be examined by ICC (1, 1). Instead, ICC (1, 4) was high enough and therefore the changes in the monthly severity score had high reliability. Because there is no other method to capture data on overuse injuries based on the ‘any physical complaint’ definition, we could not examine the criterion-related validity of the translated questionnaire. However, the results of our research on swimming athletes demonstrated that we could successfully capture data on all physical complaints, with a high response rate.

The results of this study demonstrated that the prevalence of overuse injuries varied by weeks, and the average rate of overuse injuries was 49.1 whereas that of the substantial injuries was 10.9. In the previous study, although the questionnaire was different from the present study, the prevalence of overuse injury in swimmers was also high. [13] In the previous studies related to other sports using the OSTRC questionnaire, the prevalence of injuries in handball, orienteering, tennis, and volleyball was 22 on an average, and the prevalence of the substantial problems was 9.[8] Clarsen et al.[5] reported that an average of 39 of athletes reported having overuse injuries and 13 reported having substantial injuries. The present study of female college swimmers demonstrated a higher prevalence of these injuries than the previous studies did, although these swimmers had comparable prevalence of substantial injuries with previous studies. These results followed another study, [14] which reported that the injury rate for female swimmers was higher than that in other NCAA sports. We presume that the more participants of the present study tended to continue practicing and competing, even with some body problems compared to those of the other population.

In each anatomical area, overuse and substantial injuries occurred most frequently in the lower back. In the previous studies about swimmers,[4, 15] problems in the shoulder occurred most frequently. This may be due to the definition of injury. The definition “any physical complaint” was used in this study, whereas the definition was “medical attention” in the previous studies.[4, 15] The present study clarified the prevalence of overuse injuries of the lower back, which do not lead to medical attention. The results may be affected by the swimming strokes of the participants; however, because of the small number of participants per strokes, this factor was not revealed in the present study. On the other hand, the prevalence of the shoulder problems was less than that in the previous studies. [4, 15, 16] The reason for these results could not be clarified by the present study. In future study, the characteristics of Japanese female swimmers should be examined. The prevalence of the knee and ankle problems was not as high as that for the lower back or shoulder, but the severity of knee and ankle injuries was comparable to the severity of lower back injuries. This suggests that although the prevalence of the knee and ankle injuries was lower than lower back and shoulder, the influence of the injuries on the performance and participation in practice or game should be considered.

The prevalence of internal medicine problems, which means illness, was 1.1. In a previous study, [9] the prevalence of illness was 13. This suggests that the internal medicine problems are underestimated, when the option for internal medicine was written along with the problems of the anatomical areas in the questionnaire. The severity score of the internal medicine problems was high. This indicates that the internal medicine problems have a major influence on training or competition, although their prevalence is low. This trend is similar to that seen previously. [9]

There are some limitations in the present study. Firstly, we did not record specific diagnoses for the overuse injuries that athletes reported, although the physiotherapist interviewed them to determine the type and nature of each problem. Secondly, there is a possibility that the responses were not correct. The reported anatomical area may be incorrect, or the athletes may conceal the presence of injury. Finally, the athletes of the present study were all members of a single swimming team during half of the year and represented a small sample size. The tendency of injury in other populations of swimming athletes may be different. Moreover, although this study suggested that a modified questionnaire may be useful for other Japanese swimmers, the questionnaire was not examined for its utility in assessing Japanese athletes as a whole. In the future, an additional study should be conducted in a large population for at least a one year duration.

## Supporting information

S1 File This is the S1_Japanese_version. (PDF)
