# Supplementary material for: Japanese translation and modification of the Oslo Sports Trauma Research Centre overuse injury questionnaire to evaluate overuse injuries in female college swimmers
Source: PLoS One. 2019 Apr 15;14(4):e0215352. doi: 10.1371/journal.pone.0215352 (PMC6464216; doi:10.1371/journal.pone.0215352)
Supplement: S1 File — (PDF) [file pone.0215352.s001.pdf]

質問１
先週１週間に身体上の症状により、通常の練習や試合への参加に影響が出ましたか？

a. 症状なく、全ての練習や試合に参加することができた。
b. 症状はあったが、全ての練習や試合に参加することができた。
c. 症状があり、練習や試合への参加を減らした。
d. 症状があり、練習や試合を行うことが不可能だった。

質問２
先週１週間に身体上の症状により、どの程度、練習量を減らしましたか？

a. 全く減らさなかった。
b. 少し減らした。
c. 半分程度減らした。
d. かなり減らした。
e. 練習や試合が全くできなかった。

質問３
先週１週間に身体上の症状が、どの程度、パフォーマンスに影響しましたか？

a. 全く影響しなかった。
b. 少し影響した。
c. ある程度影響した。
d. かなり影響した。
e. 全く練習や試合ができない程、影響した。

質問４
先週１ 週間に経験した身体の痛みはどの程度でしたか?？

a. 全く痛みはなかった。
b. 少し痛みがあった。
c. 中程度の痛みがあった。
d. かなりの痛みがあった。

質問 5

上記の身体上の問題で最も影響した部位はどこですか?

- ☐ 肩
- ☐ 肘・上腕
- ☐ 手首・前腕
- ☐ 指
- ☐ 背部・腰部
- ☐ 骨盤・臀部
- ☐ 股関節・大腿
- ☐ 膝
- ☐ 下腿
- ☐ 足首
- ☐ 足部
- ☐ 内科的問題

質問 6

上記の身体上の問題は新しく生じた問題ですか?

- ☐ はい，新しく生じた問題です.
- ☐ いいえ，以前から続く問題です.

質問 7

他の部位で影響した部位があれば選択して下さい.

- ☐ 肩
- ☐ 肘・上腕
- ☐ 手首・前腕
- ☐ 指
- ☐ 背部・腰部
- ☐ 骨盤・臀部
- ☐ 股関節・大腿
- ☐ 膝
- ☐ 下腿
- ☐ 足首
- ☐ 足部
- ☐ 内科的問題
